# Productivity loss and indirect costs associated with cardiovascular events and related clinical procedures

**DOI:** 10.1186/s12913-015-0925-x

**Published:** 2015-06-25

**Authors:** Xue Song, Ruben G.W. Quek, Shravanthi R. Gandra, Katherine A. Cappell, Robert Fowler, Ze Cong

**Affiliations:** Truven Health Analytics, Ann Arbor, MI USA; Amgen, Thousand Oaks, CA USA; Onyx Pharmaceuticals, Inc., South San Francisco, CA, USA

**Keywords:** Indirect cost, Cardiovascular diseases, Absenteeism, Short-term disability

## Abstract

**Background:**

The high acute costs of cardiovascular disease and acute cardiovascular events are well established, particularly in terms of direct medical costs. The costs associated with lost work productivity have been described in a broad sense, but little is known about workplace absenteeism or short term disability costs among high cardiovascular risk patients. The objective of this study was to quantify workplace absenteeism (WA) and short-term disability (STD) hours and costs associated with cardiovascular events and related clinical procedures (CVERP) in United States employees with high cardiovascular risk.

**Methods:**

Medical, WA and/or STD data from the Truven Health MarketScan® Research Databases were used to select full-time employees aged 18–64 with hyperlipidemia during 2002–2011. Two cohorts (with and without CVERP) were created and screened for medical, drug, WA, and STD eligibility. The CVERP cohort was matched with a non-CVERP cohort using propensity score matching. Work loss hours and indirect costs were calculated for patients with and without CVERP and by CVERP type. Wages were based on the 2013 age-, gender-, and geographic region-adjusted wage rate from the United States Bureau of Labor Statistics.

**Results:**

A total of 5,808 WA-eligible, 21,006 STD-eligible, and 3,362 combined WA and STD eligible patients with CVERP were well matched to patients without CVERP, creating three cohorts of patients with CVERP and three cohorts of patients without CVERP. Demographics were similar across cohorts (mean age 52.2-53.1 years, male 81.3-86.8 %). During the first month of follow-up, patients with CVERP had more WA/STD-related hours lost compared with patients without CVERP (WA-eligible: 23.4 more hours, STD-eligible: 51.7 more hours, WA and STD-eligible: 56.3 more hours) (*p* < 0.001). Corresponding costs were $683, $895, and $1,119 higher, respectively (*p* < 0.001). Differences narrowed with longer follow-up. In the first month and year of follow-up, patients with coronary artery bypass graft experienced the highest WA/STD-related hours lost and costs compared with patients with other CVERP.

**Conclusions:**

CVERP were associated with substantial work loss and indirect costs. Prevention or reduction of CVERP could result in WA and STD-related cost savings for employers.

**Electronic supplementary material:**

The online version of this article (doi:10.1186/s12913-015-0925-x) contains supplementary material, which is available to authorized users.

## Background

Cardiovascular disease (CVD) is responsible for 30 % of deaths worldwide [1] and is the primary cause of mortality in the United States. [2] CVD affects one third of adults in the United States, [3] with the prevalence projected to increase to 40.5 % of the population by 2030 [2]. A large number of patients with a high level of cardiovascular (CV) risk are unable to adequately control their low-density lipoprotein cholesterol (LDL-C) levels, increasing their susceptibility to CVD [4].

The economic burden of CVD in the United States is substantial [1–3, 5–9]. In order to obtain a complete picture of the societal costs of a health condition or health risk, both the direct and indirect costs should be considered [10, 11]. The direct costs of a condition include the costs related to the diagnosis and treatment of the condition. The indirect costs include lost work productivity, as well as loss of future productivity due to reduced employment, un-employment, or premature death. The productivity losses of caregivers are also considered to be indirect costs of a condition. Total CVD-related direct medical costs accounted for an estimated 17 % of overall health expenditures in the United States in 2005 dollars [5]. In 2010, CVD-related direct medical costs in the United States were $273 billion and CVD-related indirect costs were $172 billion [2]. By 2030, the indirect costs of CVD are expected to increase to $276 billion [2].

Estimating the overall societal costs of CVD taking into account both direct and indirect costs from multiple perspectives (*e.g.*, patients, caregivers, employers), is clearly important. However, there is also a need for estimates of the costs of CVD from more focused perspectives, such as from an employer perspective. In the face of rising healthcare costs in the United States, and the substantial proportion of those costs for which employers are responsible in the form of health insurance premiums and disability benefits, there is a growing body of literature addressing the impact of health risk factors and specific health conditions on employees’ healthcare costs. In terms of direct healthcare costs, multiple studies have demonstrated the financial burden on employers resulting from employees’ specific illnesses [12–14] or modifiable health risk factors [15–18]. Direct healthcare costs have been found to be correlated with the type and number of health risks [16, 18, 19].

Additional research has evaluated the impact of employee health on workplace productivity. Several studies have demonstrated associations between the presence of specific health conditions, [8, 12–14] or health risk factors [9, 20–22] and increased employee absence or disability. The presence of multiple risk factors has also been shown to affect the magnitude of productivity losses [23]. Finally, several repeated measures studies on the same individuals have shown that changes in health risks over time may impact productivity; specifically, that increases in health risks decrease productivity [24] and that reductions in health risks increase work productivity [25].

While employers may recognize the impact of CVD on employee productivity, the existing literature on the indirect costs of CVD in the United States is somewhat limited, and a precise and current quantification of the magnitude of the problem is currently lacking. In a study that is now over a decade old, using a number of different data sources, Goetzel and colleagues estimated that indirect costs to employers per employee with heart disease ranged from $222 to $3,301 per year, illustrating the challenges in developing precise estimates of indirect costs [9]. In another study, also ten years old, Wu *et al.* estimated $2,134 in annual excess indirect costs to employers in patients with atrial fibrillation [7]. In a more recent study, Johnston *et al.* estimated an annual incremental impact of acute coronary syndrome (ACS) on workplace absenteeism and short-term disability of $465 and $999, respectively [8]. Although these studies quantified CVD-related indirect costs, they examined productivity loss in very specific, narrowly defined CVD populations. Furthermore, the cost estimates produced by these studies are outdated. Additionally, the existing literature does not differentiate between the acute versus long-term impact of CVD on work productivity loss.

The high short-term costs of CVD and acute CV events are well established, particularly with respect to direct medical costs. The costs to employers associated with lost work productivity have been described in a broad sense, but little is known about workplace absenteeism or short term disability costs among high CV risk patients. Therefore, the objective of the current study was to examine, from the perspective of the employer, the productivity losses and indirect costs associated with a wide range of CV events and related clinical procedures (CVERP) among patients at high cardiovascular risk, as determined by a diagnosis of hyperlipidemia. This study provides a detailed and contemporary estimate of the impact of high cardiovascular risk on indirect costs to employers, and the potential for cost savings as a result of CVERP prevention.

## Methods

### Data sources

Data were extracted from the Truven Health MarketScan® Commercial Claims and Encounters (Commercial) and Health and Productivity Management (HPM) Databases. Data from these databases are the basis of over 700 peer-reviewed articles published in clinical, health policy, and health economics journals covering a wide range of therapeutic areas [14, 26–29].

The Commercial Database contains privately insured inpatient and outpatient medical and outpatient prescription drug claims, providing detailed utilization and cost data [30], In 2011, there were approximately 35 million enrollees in the database. Healthcare is provided under a variety of health plans with fee-for-service and capitated payment arrangements, including preferred provider organizations, point of service plans, indemnity plans, and health maintenance organizations. More than 100 employers and 15 health plans contribute data to the Commercial Database.

The HPM Database contains workplace absenteeism (WA), short-term disability (STD), and workers’ compensation data for a subset of enrollees in the Commercial Database. Approximately 70 employers contribute data to the HPM Database. The HPM data are linkable to the corresponding medical and pharmacy claims data for these employees.

Data in both the Commercial and HPM Databases are de-identified, and are in compliance with the Health Insurance Portability and Accountability Act (HIPAA) regulations. Thus, Institutional Review Board approval was not required to conduct this study.

### Patient selection

Patients were initially selected for the study sample based on the presence of at least one inpatient or two outpatient medical claims for hyperlipidemia [International Classification of Diseases, Ninth Revision, Clinical Modification (ICD-9-CM) diagnosis codes 272.0-272.4] or at least one outpatient prescription claim for lipid-lowering therapy between 2002 and 2011. Qualifying outpatient diagnosis claims were required to be at least 30 days but no more than 365 days apart, thus increasing the likelihood that the outpatient claims represented a true hyperlipidemia diagnosis.

Patients were subsequently stratified by the presence or absence of CVERP, either before or after the hyperlipidemia diagnosis or medication prescription, between 2002 and 2011. CVERP was defined as myocardial infarction (MI), ischemic stroke, hospitalization with unstable angina as primary discharge diagnosis, revascularization [coronary artery bypass graft (CABG) or percutaneous coronary intervention (PCI)], hospitalization with heart failure (HF) as primary discharge diagnosis, and hospitalization with transient ischemic attack (TIA) as primary discharge diagnosis. For patients with CVERP, the index date was defined as the date of the first CVERP. Patients with more than one CVERP type on the index date were hierarchically assigned a specific type in the following order: MI, ischemic stroke, unstable angina, revascularization, HF, or TIA (diagnosis and procedure codes used to identify events are available upon request). Patients with rule-out or diagnostic only claims for these events were excluded. For each patient with CVERP, the number of days between the index date and the date of the first claim indicating hyperlipidemia or lipid-lowering therapy was calculated, and an “interval pool” containing these values was created. For each patient without CVERP, a random number was selected from the interval pool and added to the date of the first claim for hyperlipidemia or lipid-lowering therapy, with the resulting date assigned as the index date [31, 32]. After this assignment, patients with and without CVERP had similar distributions of the number of days between the first claim indicating hyperlipidemia or lipid-lowering therapy and the index date. Because of the study eligibility requirements, index dates for the cohorts both with and without CVERP occurred between January 1, 2003 and November 30, 2011.

Patients with and without CVERP were additionally screened to include patients aged 18–64 years with continuous medical and drug coverage for at least 12 months prior to and at least one month after the index date. Patients were further screened to include only those who were full-time employees for the 12 months prior to and at least one month after the index date and who were not pregnant for the 12 months prior to the index date through the entire follow-up period. The follow-up period ended at the earliest of inpatient death, end of full-time employment, end of continuous enrollment, end of WA or STD benefit eligibility, or end of the study period (*i.e.*, December 31, 2011).

**Fig. 1:**
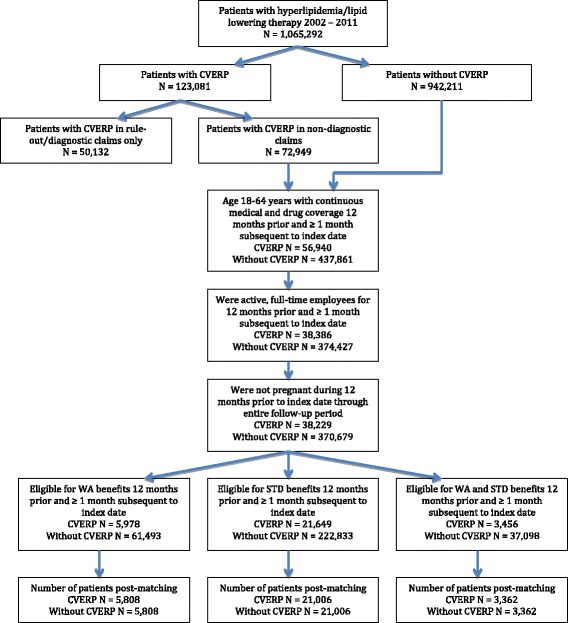
Patient selection. CVERP: cardiovascular events and related clinical procedures; STD: short-term disability; WA: workplace absenteeism

Finally, patients with CVERP and without CVERP were screened for WA and STD benefit eligibility for a minimum of 12 months prior to and one month after the index date, creating cohorts based on WA eligibility, STD eligibility, and eligibility for both benefits. Within each of these eligibility cohorts, patients with CVERP and without CVERP were matched via propensity score matching to create a total of six study cohorts. The patient selection process is presented in Fig. 1.

### Covariates

Demographic variables measured on the index date are shown in Table 1. The clinical variables, evaluated for the 12 months prior to the index date, are listed in Table 2.

**Table 1:** Demographic characteristics of propensity score matched cohorts

Characteristic	Patients with WA eligibility					Patients with STD eligibility					Patients with WA and STD eligibility				
	With CVERP		Without CVERP		Std Diff (%)	With CVERP		Without CVERP		Std Diff (%)	With CVERP		Without CVERP		Std Diff (%)
	N = 5,808		N = 5,808			N = 21,006		N = 21,006			N = 3,362		N = 3,362		
	n/mean	%/SD	n/mean	%/SD		n/mean	%/SD	n/mean	%/SD		n/mean	%/SD	n/mean	%/SD	
Age (mean, SD)	52.7	6.5	52.3	6.8	0.1	52.8	6.8	52.2	7.2	0.1	53.1	6.5	52.6	7.0	0.1
Age Group (years) (n, %)															
18-44	643	11.1	615	10.6	1.6	2524	12.0	2528	12.0	0.1	346	10.3	335	10.0	1.1
45-54	2,632	45.3	2,632	45.3	0.0	9,012	42.9	9,100	43.3	0.8	1,472	43.8	1,458	43.4	0.8
55-64	2,533	43.6	2,561	44.1	1.0	9,470	45.1	9,378	44.6	0.9	1,544	45.9	1,569	46.7	1.5
Male (n, %)	4,974	85.6	5,012	86.3	1.9	17,294	82.3	17,085	81.3	2.6	2,883	85.8	2,917	86.8	2.9
Female (n, %)	834	14.4	796	13.7	1.9	3,712	17.7	3,921	18.7	2.6	479	14.2	445	13.2	2.9
Geographic Region (n, %)															
Northeast	954	16.4	906	15.6	2.3	3428	16.3	3542	16.9	1.5	643	19.1	637	18.9	0.5
North Central	1,079	18.6	1,087	18.7	0.4	7,461	35.5	7,366	35.1	0.9	526	15.6	519	15.4	0.6
South	2,742	47.2	2,803	48.3	2.1	7,287	34.7	7,268	34.6	0.2	1,272	37.8	1,291	38.4	1.2
West	1,032	17.8	1,008	17.4	1.1	2,774	13.2	2,768	13.2	0.1	921	27.4	914	27.2	0.5
Unknown	1	0.0	4	0.1	2.5	56	0.3	62	0.3	0.5	0	0.0	1	0.0	n/a
Health Plan Type (n, %)															
Preferred provider organization	3,042	52.4	3,080	53.0	1.3	13,199	62.8	13,254	63.1	0.5	1,942	57.8	1,913	56.9	1.7
Health maintenance organization	833	14.3	826	14.2	0.3	2,592	12.3	2,595	12.4	0.0	398	11.8	436	13.0	3.4
Point of Service	1,704	29.3	1,669	28.7	1.3	2,211	10.5	2,411	11.5	3.0	885	26.3	883	26.3	0.1
Other/Unknown	229	3.9	233	4.0	0.4	3,004	14.3	2,746	13.1	3.6	137	4.1	130	3.9	1.1
Length of Follow-up															
Days (mean, SD)	650	392	717	374	0.2	559	370	602	369	0.1	560	374	622	364	0.2
≥1 year (n, %)	3,905	67.2	4,332	74.6	16.2	12,632	60.1	13,527	64.4	8.8	2,030	60.4	2,249	66.9	13.6
≥2 years (n, %)	2,672	46.0	3,053	52.6	13.1	7,196	34.3	8,038	38.3	8.3	1,184	35.2	1,364	40.6	11.1
≥3 years (n, %)	1,826	31.4	2,101	36.2	10.0	3,984	19.0	4,635	22.1	7.7	622	18.5	759	22.6	10.1

CVERP: cardiovascular events and related clinical procedures; SD: standard deviation; STD: short-term disability; Std Diff: standardized difference; WA: workplace absenteeism

**Table 2:** Clinical characteristics of propensity score matched cohorts

Characteristic	Patients with WA eligibility					Patients with STD eligibility					Patients with WA and STD eligibility				
	With CVERP		Without CVERP		Std Diff (%)	With CVERP		Without CVERP		Std Diff (%)	With CVERP		Without CVERP		Std Diff (%)
	N = 5,808		N = 5,808			N = 21,006		N = 21,006			N = 3,362		N = 3,362		
	n/mean	%/SD	n/mean	%/SD		n/mean	%/SD	n/mean	%/SD		n/mean	%/SD	n/mean	%/SD	
1-year Pre-Index Period Deyo Charlson Comorbidity Index (mean, SD)	0.6	1.1	0.6	1.3	0.004	0.7	1.2	0.7	1.4	0.043	0.7	1.1	0.7	1.4	0.01
1-year Pre-Index Period Comorbid Conditions (n, %)															
Diabetes	1,108	19.1	989	17.0	5.3	4,172	19.9	4,035	19.2	1.6	690	20.5	689	20.5	0.1
Hypertension	2,207	38.0	2,223	38.3	0.6	8,153	38.8	7,980	38.0	1.7	1,345	40.0	1,351	40.2	0.4
Stable angina	343	5.9	313	5.4	2.2	1,152	5.5	1,098	5.2	1.1	173	5.1	154	4.6	2.6
Cancer	201	3.5	361	6.2	12.9	742	3.5	1,359	6.5	13.5	118	3.5	206	6.1	12.2
Respiratory disease	1,906	32.8	1,923	33.1	0.6	7,015	33.4	7,133	34.0	1.2	1,098	32.7	1,102	32.8	0.3
Renal disease	162	2.8	161	2.8	0.1	675	3.2	598	2.8	2.1	100	3.0	109	3.2	1.5
Liver disease	99	1.7	106	1.8	0.9	379	1.8	466	2.2	3.0	63	1.9	54	1.6	2.0
Peripheral vascular disease	159	2.7	87	1.5	8.6	733	3.5	551	2.6	5.0	93	2.8	47	1.4	9.6
Psychiatric disorders	641	11.0	580	10.0	3.4	2,735	13.0	2,850	13.6	1.6	405	12.0	392	11.7	1.2
Abdominal aortic aneurysm	20	0.3	17	0.3	0.9	109	0.5	96	0.5	0.9	14	0.4	14	0.4	0.0
Metabolic syndrome	21	0.4	20	0.3	0.3	74	0.4	119	0.6	3.2	10	0.3	17	0.5	3.3
Obesity of overweight	107	1.8	114	2.0	0.9	503	2.4	509	2.4	0.2	77	2.3	86	2.6	1.7
Arrhythmias	414	7.1	430	7.4	1.1	1,567	7.5	1,526	7.3	0.7	242	7.2	232	6.9	1.2
Baseline Hours Lost															
Monthly hours lost due to WA/STD/WA and STD during the 1-year Pre-Index Period (mean, SD)	20.5	18.9	20.5	21.7	0.002	4.7	20.0	5.4	23.0	0.03	23.3	19.4	23.2	21.3	0.01

CVERP: cardiovascular events and related clinical procedures; SD: standard deviation; STD: short-term disability; Std Diff: standardized difference; WA: workplace absenteeism

### Outcomes

#### Workplace absenteeism

For patients with WA benefit eligibility, the number and percentage of patients with WA, the hours lost due to WA [per patient per month (PPPM)], and the PPPM indirect costs associated with WA were reported. These endpoints were measured during the 12-month pre-period, the first month of the follow-up period, the first year of follow-up in patients with ≥ 1 year of follow-up, the second year of follow-up in patients with ≥ 2 years of follow-up, and the third year of follow-up in patients with ≥ 3 years of follow-up.

Indirect costs associated with WA were calculated by multiplying the number of hours absent during the specified time periods with an average hourly wage. Wages were based on the 2013 age-, gender-, and geographic region-adjusted wage rate from the United States Bureau of Labor Statistics (BLS) [33]. Due to the small sample size of patients within each industry in the study population, industry-specific wage rates were not applied.

### Short-term disability

For patients with STD benefit eligibility, the number and percentage of patients with STD, the PPPM hours lost due to STD, and the PPPM indirect costs associated with STD were reported. Because employers typically pay 60 % of wages as part of an STD benefit, [34] this percentage was applied to the BLS daily wage rate when calculating the indirect costs associated with STD. The reporting time periods were the same as those used for WA.

### Workplace absenteeism and short-term disability

For patients with both WA and STD benefit eligibility, productivity losses and indirect costs due to WA and STD combined were measured during the time periods described above.

Among patients with CVERP, PPPM hours and indirect costs were also reported stratified by CVERP type for the first month of the follow-up period, as well as for the first year of follow-up in patients with ≥ 1 year of follow-up in the WA, STD, and combined benefit eligibility cohorts.

### Propensity score matching

As this is an observational study in which randomization of patients was not possible, propensity score matching [35, 36] was used to ensure a similar distribution of characteristics that may influence the development of CVERP and potentially confound productivity loss between patients with and without CVERP. The intention of propensity score matching is to develop similar cohorts of patients who differ only with respect to the exposure of interest, which in this analysis was CVERP. A logistic regression model was used to predict the probability (*i.e.*, generate a propensity score) that a patient would experience CVERP based on observed characteristics. Matching factors were pre-period work loss as measured by WA or STD hours, age (<45, 45–54, 55–64), gender, health plan, geographic region, Deyo Charlson comorbidity index (DCI), [37] diabetes, hypertension, cancer, respiratory disease, renal disease, liver disease, psychiatric disorders, obesity, and metabolic syndrome. Due to the lack of information on characteristics of the employers, matching was conducted based on patient characteristics only.

Patients with CVERP were matched with the pool of patients without CVERP using the model-generated propensity score. In order to achieve better external validity, the nearest neighbor with 1:1 and caliper matching technique [38] was used, as this allowed inclusion of every observation from the smaller sample sized cohort to the extent possible. To evaluate whether patients with and without CVERP were matched successfully, the standardized difference was calculated for each of the matching factors and compared with the recommended threshold of 10 % [38].

### Analyses

Descriptive analyses were conducted on all patient characteristics, WA and STD hours, and associated indirect costs, separately for patients with and without CVERP. Continuous measures were summarized as means and standard deviations. Categorical measures were summarized as counts and percentages. Statistical tests of significance for differences in WA and STD hours and indirect costs between patients with and without CVERP were performed. Chi-square tests were used to evaluate the statistical significance of differences for categorical variables. Fisher’s exact test was used to evaluate the statistical significance for categorical variables with rare events. To evaluate the statistical significance of differences for normally distributed continuous variables, t-tests were used.

## Results

### Patient selection

A total of 1,065,292 patients with a diagnosis of hyperlipidemia or use of lipid-lowering medications were selected for the time period 2002 to 2011 (Fig. 1). Among these patients, 38,229 experienced CVERP and met all remaining study inclusion criteria. Further screening for patients eligible for WA, STD, and both benefits resulted in 5,978, 21,649, and 3,456 patients, respectively. After propensity score matching, 5,808, 21,006 and 3,362 matched pairs of patients with and without CVERP were available for respective analysis of WA, STD and combined benefits.

### Demographic and clinical characteristics

Patients were well matched on age, gender, geographic region, and health plan type (Table 1) and on the DCI (Table 2). Mean age ranged from 52.2-53.1 years. The cohorts were predominantly male (81.3-86.8 %), with patients tending to reside in the South (34.6-48.3 %) and North (34.3-51.9 %) regions of the United States. The DCI was similar across cohorts (0.6-0.7).

Hypertension and respiratory disease were prevalent across cohorts, with 38.0-40.2 % of patients having a claim for hypertension and 32.7-34.0 % of patients having a claim for respiratory disease in the 12 months prior to the index date. In the WA eligibility and STD eligibility cohorts, the standardized differences were less than ten for all comorbidities except cancer, indicating that the CVERP and non-CVERP cohorts were well matched.

**Fig. 2:**
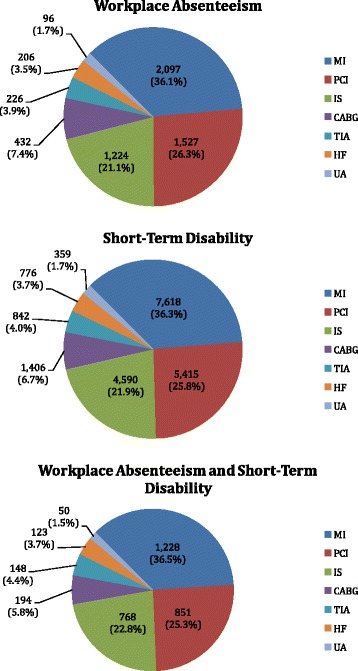
Number and Percentage of Patients by CVERP Type at Index. CVERP: cardiovascular events and related clinical procedures; CABG: coronary artery bypass graft; HF: heart failure; IS: ischemic stroke; MI: myocardial infarction; PCI: percutaneous coronary intervention; TIA: transient ischemic attack; UA: unstable angina

Figure 2 presents the distribution of CVERP type on the index date. MI occurred in 36.1-36.5 % of patients on the index date. More than 80 % (83.5-84.7 %) of patients experienced MI, PCI, or ischemic stroke on the index date.

### Productivity loss and indirect costs in patients with and without CVERP

#### Workplace absenteeism

Patients with CVERP had significantly higher PPPM hours and indirect costs due to WA compared with patients without CVERP during the first month and the first year of follow-up (Table 3). The difference was the largest in the first month of follow-up, with patients who experienced CVERP having 43.0 h and $1,267 in costs, compared with 19.7 h and $585 in costs for the patients without CVERP. In the first year of follow-up, patients with CVERP had 23.1 h and $698 in costs, while patients without CVERP had 19.8 h and $597 in costs. Among those with at least two or three years of follow-up, patients with and without CVERP did not differ significantly in PPPM WA hours or costs during the second and third years of follow-up.

**Table 3:** WA productivity loss and indirect costs in propensity score matched cohorts with WA eligibility

Measure	Patients with WA eligibility				
	With CVERP		Without CVERP		*p*-value
	N = 5,808		N = 5,808		
	n/mean	%/SD	n/mean	%/SD	
During the 1st month of follow-up:					
Patients reporting WA (n, %)	4,011	69.1	3,963	68.2	0.34
Monthly hours lost due to WA (mean, SD)	43.0	50.8	19.7	27.8	<.001
Indirect costs associated with WA (mean, SD)	$1,267	$1,511	$585	$849	<.001
Patients with ≥ 1 year of follow-up (n, %)	3,905	67.2	4,332	74.6	
During the 1st year of follow-up:					
Patients reporting WA (n, %)	3,370	86.3	3,742	86.4	0.92
Monthly hours lost due to WA (mean, SD)	23.1	21.2	19.8	17.3	<.001
Indirect costs associated with WA (mean, SD)	$698	$666	$597	$548	<.001
Patients with ≥ 2 years of follow-up (n, %)	2,672	46.0	3,053	52.6	
During the 2nd year of follow-up:					
Patients reporting WA (n, %)	2,255	84.4	2,643	86.6	0.02
Monthly hours lost due to WA (mean, SD)	19.2	16.1	19.4	16.5	0.62
Indirect costs associated with WA (mean, SD)	$565	$483	$573	$508	0.54
Patients with ≥ 3 years of follow-up (n, %)	1,826	31.4	2,101	36.2	
During the 3rd year of follow-up:					
Patients reporting WA (n, %)	1,552	85.0	1,788	85.1	0.92
Monthly hours lost due to WA (mean, SD)	19.0	15.8	19.1	17.0	0.86
Indirect costs associated with WA (mean, SD)	$548	$465	$551	$491	0.84

CVERP: cardiovascular events and related clinical procedures; SD: standard deviation; WA: workplace absenteeism

### Short-term disability

STD hours and costs were significantly higher in patients with CVERP compared with patients without CVERP for all time periods (Table 4). In the first month of follow-up, patients with CVERP had 57.7 h and $996 in costs, while patients without CVERP had 6.0 h and $102 in costs. Differences in monthly hours and costs between patients with and without CVERP were 51.7 h/$895, 12.7 h/$221, 1.9 h/$34, and 2.4 h/$40, for the first month of follow-up and during the first, second, and third years of follow-up, respectively. While a trend of decreasing productivity loss from the acute period (one month) to longer time periods (one, two, and three years) was observed, it is worth noting that only a subset of patients in the one-month analysis who had at least one, two, or three years of follow-up was eligible for the analyses involving longer time periods. The number of patients with at least one, two, and three years of follow-up are reported in Table 4.

**Table 4:** STD Productivity Loss and Indirect Costs in Propensity Score Matched Cohorts with STD Eligibility

Measure	Patients with STD Eligibility				
	With CVERP		Without CVERP		*p*-value
	N = 21,006		N = 21,006		
	n/mean	%/SD	n/mean	%/SD	
During the 1st month of follow-up:					
Patients reporting STD (n, %)	8,764	41.7	1,011	4.8	<.001
Monthly hours lost due to STD (mean, SD)	57.7	74.6	6.0	29.7	<.001
Indirect costs associated with STD (mean, SD)	$996	$1,309	$102	$506	<.001
Patients with ≥ 1 year of follow-up (n, %)	12,632	60.1	13,527	64.4	
During the 1st year of follow-up:					
Patients reporting STD (n, %)	5,890	46.6	1,406	10.4	<.001
Monthly hours lost due to STD (mean, SD)	16.3	30.8	3.6	16.7	<.001
Indirect costs associated with STD (mean, SD)	$281	$529	$60	$278	<.001
Patients with ≥ 2 years of follow-up (n, %)	7,196	34.3	8,038	38.3	
During the 2nd year of follow-up:					
Patients reporting STD (n, %)	1,031	14.3	734	9.1	<.001
Monthly hours lost due to STD (mean, SD)	4.7	18.0	2.7	13.3	<.001
Indirect costs associated with STD (mean, SD)	$79	$308	$45	$219	<.001
Patients with ≥ 3 years of follow-up (n, %)	3,984	19.0	4,635	22.1	
During the 3rd year of follow-up:					
Patients reporting STD (n, %)	536	13.5	364	7.9	<.001
Monthly hours lost due to STD (mean, SD)	4.4	17.9	2.1	10.8	<.001
Indirect costs associated with STD (mean, SD)	$73	$298	$34	$177	<.001

CVERP: cardiovascular events and related clinical procedures; SD: standard deviation; STD: short-term disability

### Workplace absenteeism and short-term disability

In patients with both WA and STD benefits, productivity losses and indirect costs were consistently greater among patients who experienced CVERP compared with those without CVERP, although the differences were not uniformly statistically significant (Table 5). The differences were significant in the first month of follow-up, with 56.3 more hours and $1,119 in additional costs for patients with CVERP, and in the first year of follow-up (for a subset of patients with at least one year of follow-up), with 13.5 more hours and $237 in additional costs for patients with CVERP.

**Table 5:** WA and STD Productivity Loss and Indirect Costs in Propensity Score Matched Cohorts with WA and STD Eligibility

Measure	Patients with WA and STD Eligibility				
	With CVERP		Without CVERP		*p*-value
	N = 3,362		N = 3,362		
	n/mean	%/SD	n/mean	%/SD	
During the 1st month of follow-up:					
Patients reporting WA/STD (n, %)	2,980	88.6	2,591	77.1	<.001
Monthly hours lost due to WA/STD (mean, SD)	78.7	63.5	22.4	30.3	<.001
Indirect costs associated with WA/STD (mean, SD)	$1,744	$1,293	$624	$785	<.001
Patients with ≥ 1 year of follow-up (n, %)	2,030	60.4	2,249	66.9	
During the 1st year of follow-up:					
Patients reporting WA/STD (n, %)	1,917	94.4	2,093	93.1	0.07
Monthly hours lost due to WA/STD (mean, SD)	35.0	29.3	21.4	14.1	<.001
Indirect costs associated with WA/STD (mean, SD)	$847	$572	$610	$374	<.001
Patients with ≥ 2 years of follow-up (n, %)	1,184	35.2	1,364	40.6	
During the 2nd year of follow-up:					
Patients reporting WA/STD (n, %)	1,088	91.9	1,294	94.9	0.002
Monthly hours lost due to WA/STD (mean, SD)	22.2	18.0	21.2	13.7	0.12
Indirect costs associated with WA/STD (mean, SD)	$590	$389	$590	$337	0.99
Patients with ≥ 3 years of follow-up (n, %)	622	18.5	759	22.6	
During the 3rd year of follow-up:					
Patients reporting WA/STD (n, %)	581	93.4	723	95.3	0.14
Monthly hours lost due to WA/STD (mean, SD)	21.3	16.2	20.7	12.9	0.44
Indirect costs associated with WA/STD (mean, SD)	$561	$359	$568	$305	0.68

CVERP: cardiovascular events and related clinical procedures; SD: standard deviation; STD: short-term disability; WA: workplace absenteeism

### Productivity loss and indirect costs by CVERP type

**Fig. 3:**
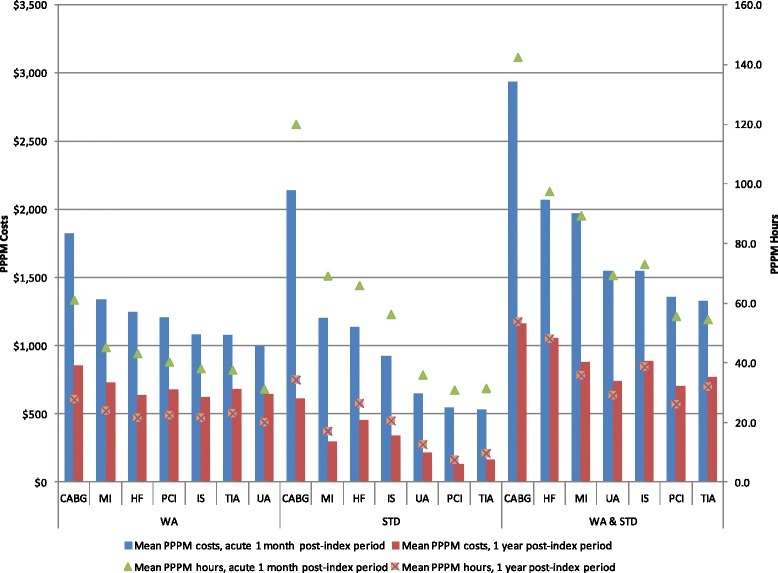
WA and STD Hours and Costs by CVERP Type. CABG: coronary artery bypass graft; HF: heart failure; IS: ischemic stroke; MI: myocardial infarction; PCI: percutaneous coronary intervention; PPPM: per patient per month; STD: short-term disability; TIA: transient ischemic attack; UA: unstable angina; WA: workplace absenteeism

In the WA, STD, and combined benefit eligibility cohorts, patients with CABG as their CVERP type experienced the highest PPPM costs both in the first month and in the first year of follow-up (Fig. 3). During the first month of follow-up, MI and HF were associated with the second and third highest costs, respectively, in the WA and STD cohorts. In the combined eligibility cohort, HF costs were higher than MI costs. For the first year of follow-up, MI and TIA patients with WA eligibility had the second and third highest costs, respectively. In the STD and combined cohorts, HF patients and ischemic stroke patients had the second and third highest costs, respectively. During the first month of follow-up, hours lost to WA, STD, or combined benefits ranged from 31–61, 31–120, and 55–143 h, respectively.

### Productivity loss and indirect costs by number of CVERP

For the first month of follow-up, WA hours were 37.9, 46.4, and 49.4, and WA costs were $1,133, $1,365, and $1,457 for patients with 1, 2, and ≥ 3 CVERP, respectively; STD hours were 36.5, 49.5, and 86.6, and STD costs were $639, $857, and $1,497, respectively (data available in Additional files 1 and 2). The number of work hours lost and the corresponding indirect costs consistently increased for patients with greater numbers of CVERP during the first, second, and third years of follow-up.

## Discussion

To our knowledge, this is the first study to examine the cost of workplace absenteeism in high cardiovascular risk patients with a range of CV events and related procedures over multiple years of follow-up. Patients with CVERP experienced significant incremental WA and STD hours and associated costs compared with patients without CVERP. Although the differences were the greatest during the first month following the event, the impact of CVERP was still observed during the first year of follow-up for WA and for up to three years for STD. Productivity losses and indirect costs were highest in patients with CABG and increased with the number of CVERP.

Several studies have evaluated productivity losses resulting from a number of non-CV-related health conditions [39–41]. Analyses of work loss specifically in patients with CVD have typically focused on specific CV conditions. For example, in an evaluation of indirect costs in patients with ACS, Johnston *et al.* found that these patients incurred 250 annual mean WA hours (20.8 h per month) and 22 annual mean STD days (14.7 h monthly, assuming 8 h days) [8]. This is consistent with our findings in the first year of follow-up of 23.9 WA PPPM hours for patients with MI and 20.1 WA PPPM hours for patients with unstable angina as their CVERP types, and of 17.1 and 12.7 STD PPPM hours for MI and unstable angina, respectively.

Data on the direct costs associated with CVD are more readily available in the published literature than are those on indirect costs. An analysis of hospitalizations for CV events found higher medical costs for MI and CABG compared with ischemic stroke, HF, angina, and TIA/other cerebrovascular accidents, [42] while an analysis of medical costs in the first month after an MI or CABG found that costs for these events were higher compared with the costs of percutaneous transluminal coronary angioplasty (PTCA), angina, and ischemic stroke [43]. Differences among types of CV events in the average duration of hospitalization have also been documented [*e.g.*, 2.71 days (angina) to 9.23 days (CABG)] [25]. Patients hospitalized for longer periods of time will incur higher medical costs, and by virtue of being hospitalized, will not be working, thus also incurring higher indirect costs. Therefore, it is likely that there is a correlation between direct and indirect costs, at least in the short-term. In our study, in the first month of follow-up, patients with CABG experienced the highest work loss and associated costs, followed by patients with MI and HF.

Medical and productivity costs may vary within a specific CV condition depending on the manner in which the patient is managed. Analyses of patients with ACS found that treatment at the initiating ACS event with non-invasive medical management resulted in the lowest direct and indirect costs, whereas treatment with an invasive CABG procedure produced the highest costs [42]. In our study, patients were hierarchically assigned to a CVERP type, based on the first occurrence of MI, ischemic stroke, unstable angina, revascularization, HF, or TIA. Thus patients assigned as MI may have had an invasive revascularization procedure later in their follow-up, which may partially explain the high WA and STD costs associated with MI. Our data support this hypothesis; more than 60 % of patients with MI at index had a revascularization procedure within three years of their index date.

In the present study, PPPM productivity losses and indirect costs were highest in the first month following CVERP. There are several possible explanations for this finding. First, many of the CVERP types were associated with a hospital admission, and some patients experienced readmissions within 30 days, thereby increasing costs associated with the first month of follow-up. Median 30-day hospital readmission rates have been estimated at 19.9 % and 24.5 % for Medicare patients with acute MI and HF, respectively [13]. Second, the difference between the first month and the one, two, and three years of follow-up may be due in part to the composition of our patient population. Because we were interested in examining indirect costs from the employer perspective, we required patients to be full-time employees and have WA and STD eligibility equivalent to the specific follow-up period. Thus, of the patients included in the first month analyses, only 31 %, 19 %, and 19 % remained for the WA, STD, and combined WA and STD analyses at three years of follow-up. By definition, patients remaining at three years of follow-up represented a working population, and patients unable to continue working and who would likely have accrued higher indirect costs in the longer follow-up periods, were not included in the analyses. Because patients who exited the workforce were not captured in the databases, productivity losses associated with CVERP may have been underestimated. Nevertheless, differences in costs were significantly higher for patients with CVERP compared with those without CVERP in the first month and the first year of follow-up for WA and STD, and additionally in the second and third years of follow-up for STD. These results indicate that this should be an area of concern for employers. In addition, the CVERP and non-CVERP cohorts were well balanced, with the only exception being that a higher proportion of the matched patients without CVERP had cancer than patients with CVERP. Despite their higher prevalence of cancer, patients without CVERP still had lower work loss and indirect costs.

With an estimated $172 billion in CVD-related indirect costs in 2010 in the United States, [2] programs that target prevention of CVD offer the potential for considerable cost savings to employers [14, 44]. A recent review of the benefits of CV risk reduction programs suggest that the return on investment to employers in terms of savings from decreases in absenteeism, workers’ compensation, disability claims, and presenteeism is substantial [30].

In patients who have already experienced or who are at high risk for experiencing a CV event, lifestyle intervention strategies alone may not be sufficient to maximally reduce CV risk [45, 46]. Current treatment guidelines in the United States recommend lipid-lowering therapy in addition to lifestyle modifications to lower LDL-C for high risk primary prevention and secondary prevention patients [47]. Lipid-lowering therapy that decreases LDL-C reduces the occurrence of both fatal and non-fatal CV events in high risk individuals [32, 48]. This study demonstrated the high productivity losses and indirect costs associated with the occurrence of CVERP, especially for CABG and MI (Fig. 3). Pharmacological treatments aimed at lowering LDL-C, via their ability to reduce the occurrence of fatal and non-fatal CV events and related procedures, such as CABG and MI, could help to reduce these substantial productivity losses and associated costs.

Several limitations of the present study should be noted. First, administrative claims were used to select patients with hyperlipidemia and CVERP and any miscoding may have resulted in the misclassification of patients, a limitation that is generalizable to all claims data analyses. Second, because the Truven Health Research Databases contain employees with commercial health insurance provided by large employers, findings from the study may not be generalizable to the entire working population in the United States, especially to employees of smaller employers. In addition, the gender distribution of the study population was skewed toward male, as CVERP are more prevalent in males. The HPM database has a balanced gender distribution and previous productivity studies (*e.g.*, asthma, rheumatoid arthritis) using the HPM database have not shown similarly skewed gender distributions. [31, 49, 50]. Third, the HPM database does not include data on presenteeism, or on-the-job productivity loss [51]. This may have resulted in underestimation of true indirect costs. Fourth, observational analyses such as the present study may be subject to residual confounding despite the use of propensity score matching, especially for characteristics that are not observed in claims data. The observed demographic and clinical characteristics were well balanced in this study, suggesting a successfully matched comparison cohort of patients with and without CVERP. However, we were not able to match the samples based on characteristics of employers, such as features of employers’ leave policies, which may have resulted in residual differences between the cohorts. During propensity score matching, the most severe patients with CVERP (<3 %) were dropped from the analyses due to the inability to find adequately matched patients without CVERP; this may also have resulted in underestimation of indirect costs. Multivariate regression analyses were also conducted to control for patients’ demographic and clinical characteristics and the estimated incremental hours associated with CVERP were consistent with the results based on propensity score matching. Fifth, because absenteeism in the database used for this study includes vacation, holidays, and jury duty in addition to sick leave, the absolute hours of WA reported are likely greater than actual leave due to illness. However, this limitation applies to both the CVERP and non-CVERP cohorts; therefore the impact should be minimal when comparing results between cohorts. As discussed above, because the study sample was drawn from large employers, the results may not reflect the productivity losses of small employers; the BLS average wage rates used in our study reflect both small and large employers, and small employers typically have a lower average wage rate [52]. Indirect costs for this study may consequently have been underestimated. Finally, this study examined productivity loss from the employers’ perspective. Future analysis that investigates the indirect cost savings from the patients’ perspective would help provide a more complete picture of indirect costs associated with CVERP.

## Conclusions

CVERP were associated with significant productivity losses and indirect costs among patients with high cardiovascular risk. Programs and/or interventions used to prevent or reduce the occurrence of CVERP in high risk patients may result in substantial cost savings for employers.

## Additional files

Additional file 1:
**WA Productivity Loss and Indirect Costs by Number of CVERPs During Follow-Up in Propensity Score Matched Cohorts with WA Eligibility.**
Additional file 2:
**STD Productivity Loss and Indirect Costs by Number of CVERPs During Follow-Up in Propensity Score Matched Cohorts with STD Eligibility.**

## Abbreviations

ACS: Acute coronary syndrome
BLS: Bureau of Labor Statistics
CABG: Coronary artery bypass graft
CVD: Cardiovascular disease
CVERP: Cardiovascular events and related clinical procedures
DCI: Deyo Charlson comorbidity index
HF: Heart failure
HIPAA: Health Insurance Portability and Accountability Act
HPM: Health and productivity management
ICD-9-CM: International Classifications of Diseases, 9th Revision, Clinical Modification
IS: Ischemic stroke
LDL-C: Low density lipoprotein cholesterol
LOS: Length of stay
MI: Myocardial infarction
PCI: Percutaneous coronary intervention
PPPM: Per patient per month
PTCA: Percutaneous transluminal coronary angioplasty
SD: Standard deviation
STD: Short-term disability
Std Diff: Standardized difference
TIA: Transient ischemic attack
UA: Unstable angina
US: United States
WA: Workplace absenteeism
