# Supplementary material for: Productivity loss and indirect costs associated with cardiovascular events and related clinical procedures
Source: BMC Health Serv Res. 2015 Jun 25;15:245. doi: 10.1186/s12913-015-0925-x (PMC4478719; doi:10.1186/s12913-015-0925-x)
Supplement: Additional file 1: — WA Productivity Loss and Indirect Costs by Number of CVERPs During Follow-Up in Propensity Score Matched Cohorts with WA Eligibility. [file 12913_2015_925_MOESM1_ESM.docx]

Patients with hyperlipidemia/lipid lowering therapy 2002 – 2011

N = 1,065,292

Patients with CVERP in rule-out/diagnostic claims only

N = 50,132

Patients with CVERP

N = 123,081

Patients with CVERP in non-diagnostic claims

N = 72,949

Patients without CVERP

N = 942,211

Age 18-64 years with continuous medical and drug coverage 12 months prior and ≥ 1 month subsequent to index date

CVERP N = 56,940

Without CVERP N = 437,861

Were active, full-time employees for 12 months prior and ≥ 1 month subsequent to index date

CVERP N = 38,386

Without CVERP N = 374,427

Were not pregnant during 12 months prior to index date through entire follow-up period

CVERP N = 38,229

Without CVERP N = 370,679

Eligible for WA benefits 12 months prior and ≥ 1 month subsequent to index date

CVERP N = 5,978

Without CVERP N = 61,493

Eligible for STD benefits 12 months prior and ≥ 1 month subsequent to index date

CVERP N = 21,649

Without CVERP N = 222,833

Eligible for WA and STD benefits 12 months prior and ≥ 1 month subsequent to index date

CVERP N = 3,456

Without CVERP N = 37,098

Number of patients post-matching

CVERP N = 5,808

Without CVERP N = 5,808

Number of patients post-matching

CVERP N = 21,006

Without CVERP N = 21,006

Number of patients post-matching

CVERP N = 3,362

Without CVERP N = 3,362
