# Supplementary material for: Productivity loss and indirect costs associated with cardiovascular events and related clinical procedures
Source: BMC Health Serv Res. 2015 Jun 25;15:245. doi: 10.1186/s12913-015-0925-x (PMC4478719; doi:10.1186/s12913-015-0925-x)
Supplement: Additional file 2: — STD Productivity Loss and Indirect Costs by Number of CVERPs During Follow-Up in Propensity Score Matched Cohorts with STD Eligibility. [file 12913_2015_925_MOESM2_ESM.docx]

Additional File 2

| STD Productivity Loss and Indirect Costs by Number of CVERPs During Follow-Up in Propensity Score Matched Cohorts with STD Eligibility | | | | | | | | | | |  |  |
| --- | --- | --- | --- | --- | --- | --- | --- | --- | --- | --- | --- | --- |
|  |  | |  |  | |  |  | |  | |  |  |
| Measure | Patients with STD Eligibility | | | | | | | | | | | |
|  | No CVERP | | | With 1 CVERP | | | With 2 CVERPs | | | With 3+ CVERPs | | |
|  | N = | 27,374 | | N = | 3,322 | | N = | 1,946 | | N = | | 9,370 |
|  | n/mean | %/SD | | n/mean | %/SD | | n/mean | %/SD | | n/mean | | %/SD |
| During the 1st month of follow-up: |  |  | |  |  | |  |  | |  | |  |
| Patients reporting STD (n, %) | 2,528 | 9.2 | | 1,019 | 30.7 | | 743 | 38.2 | | 5,485 | | 58.5 |
| Monthly hours lost due to STD (mean, SD) | 11.3 | 39.5 | | 36.5 | 62.8 | | 49.5 | 70.2 | | 86.6 | | 78.7 |
| Indirect costs associated with STD (mean, SD) | $191 | $674 | | $639 | $1,112 | | $857 | $1,237 | | $1,497 | | $1,390 |
|  |  |  | |  |  | |  |  | |  | |  |
| Patients with ≥1 year of follow-up (n,%) | 17,190 | 62.8 | | 2,055 | 61.9 | | 1,162 | 59.7 | | 5,752 | | 61.4 |
| During the 1st year of follow-up: |  |  | |  |  | |  |  | |  | |  |
| Patients reporting STD (n, %) | 2,475 | 14.4 | | 708 | 34.5 | | 496 | 42.7 | | 3,617 | | 62.9 |
| Monthly hours lost due to STD (mean, SD) | 4.8 | 19.0 | | 9.7 | 24.6 | | 11.3 | 23.9 | | 24.1 | | 35.0 |
| Indirect costs associated with STD (mean, SD) | $80 | $317 | | $168 | $421 | | $195 | $411 | | $419 | | $607 |
|  |  |  | |  |  | |  |  | |  | |  |
| Patients with ≥2 years of follow-up (n,%) | 10,022 | 36.6 | | 1,223 | 36.8 | | 659 | 33.9 | | 3,330 | | 35.5 |
| During the 2nd year of follow-up: |  |  | |  |  | |  |  | |  | |  |
| Patients reporting STD (n, %) | 976 | 9.7 | | 148 | 12.1 | | 73 | 11.1 | | 568 | | 17.1 |
| Monthly hours lost due to STD (mean, SD) | 2.9 | 13.8 | | 3.7 | 15.6 | | 3.7 | 17.4 | | 5.7 | | 20.2 |
| Indirect costs associated with STD (mean, SD) | $48 | $226 | | $63 | $270 | | $64 | $298 | | $98 | | $349 |
|  |  |  | |  |  | |  |  | |  | |  |
| Patients with ≥3 years of follow-up (n,%) | 5,701 | 20.8 | | 716 | 21.6 | | 389 | 20.0 | | 1,813 | | 19.3 |
| During the 3rd year of follow-up: |  |  | |  |  | |  |  | |  | |  |
| Patients reporting STD (n, %) | 487 | 8.4% | | 92 | 12.8% | | 51 | 13.0% | | 279 | | 15.3% |
| Monthly hours lost due to STD (mean, SD) | 2.7 | 14.3 | | 3.4 | 13.0 | | 4.8 | 18.5 | | 4.9 | | 18.5 |
| Indirect costs associated with STD (mean, SD) | $43 | $227 | | $57 | $215 | | $81 | $308 | | $84 | | $324 |
|  |  |  | |  |  | |  |  | |  | |  |

CVERP: cardiovascular events and related clinical procedures; SD: standard deviation; STD: short-term disability
